# The complete mitochondrial genome of *Penaeus semisulcatus* (Decapoda: Penaeidae)

**DOI:** 10.1080/23802359.2019.1659116

**Published:** 2019-09-27

**Authors:** Shengping Zhong, Yanfei Zhao, Guoqiang Huang, Weiling Xu

**Affiliations:** a Institute of Marine Drugs, Guangxi University of Chinese Medicine, Nanning, China;; b Key Laboratory of Marine Biotechnology, Guangxi Institute of Oceanology, Beihai, China;; c Institute of Economic Management, Beihai Vocational College, Beihai, China

**Keywords:** Mitochondrial genome, *Penaeus semisulcatus*, Decapoda

## Abstract

The green tiger prawn, *Penaeus semisulcatus*, is one of the economically important penaeid shrimp due to its larger body size compared with other shrimps in Penaeidae family. However, the taxonomic revision studies of Penaeidae have been one of the most controversial issues in recent years. Moreover, there are at least two morphotypes of the green tiger prawn, one with banded antenna, another with non-banded antenna. In this study, we report the complete mitochondrial genome of *P. semisulcatus*. The mitogenome has 16,002 base pairs (68.6% A + T content) and made up of total of 37 genes (13 protein-coding, 22 transfer RNAs and 2 ribosomal RNAs), and a control region. This study adds one more available complete mitogenomes of *Penaeus* and will provide useful genetic information for future evolutionary and taxonomic classification of Penaeidae.

Penaeidae constitute a diverse and abundant group of economically important benthic shrimps, which distributed tropical and subtropical waters around the world (Ma et al. 2011). The green tiger prawn, *P. semisulcatus*, is an ecologically and economically important penaeid shrimp throughout the Indo-west Pacific region which inhabits marine muddy and sandy bottom from 2 to 130 m depth (Hassanien and Al-Rashada 2019). While much has been studied about the physiology of Penaeidae, however, there is still considerable doubt about the taxonomic revision and evolutionary relationships in this group (Ma et al. 2009). The taxonomic status of the morphotypes of *P. semisulcatus* has been debated for there are at least two morphotypes within the green tiger prawn (Tamadoni Jahromi et al. 2019). The complete mitochondrial genome is useful molecular techniques for solving taxonomic problems (Ma et al. 2009). Here, we report the complete mitochondrial genome sequence of *P. semisulcatus*, which will provide a better insight into taxonomic classification and evolutionary relationship of Penaeidae that is the most commercially important decapods worldwide.

A tissue samples of *P. semisulcatus* from 3 individuals were collected from GuangXi province, China (Beihai, 21.451585 N, 109.331776 E), and the whole body specimen (#GQ0328) were deposited at Marine biological Herbarium, Guangxi Institute of Oceanology, Beihai, China. The total genomic DNA was extracted from the muscle of the specimens using an SQ Tissue DNA Kit (OMEGA, Guangzhou, China) following the manufacturer’s protocol. DNA libraries (350 bp insert) were constructed with the TruSeq NanoTM kit (Illumina, San Diego, CA) and were sequenced (2 × 150bp paired-end) using HiSeq platform at Novogene Company, China. Mitogenome assembly was performed by MITObim. Complete mitogenome of *Penaeus monodon* (GenBank accession number: NC_002184) was chosen as the initial reference sequence for MITObim assembly. Gene annotation was performed by MITOS.

The complete mitogenome of *P. semisulcatus* was 16,002 bp in length (GenBank accession number: MG821354), and containing the typical set of 13 protein-coding, 22 tRNA and 2 rRNA genes, and a putative control region. The overall base composition of the mitogenome was estimated to be A 33.7%, T 34.9%, C 19.0% and G 12.4%, with a high A + T content of 68.6%, which is similar, but slightly lower than *Melicertus latisulcatus* (64.7%) (Zhong et al. 2018). *Penaeus semisulcatus* and *P. monodon* shared very similar morphological characters, which has hepatic ridge on carapace, closed thelycum and brownish grey dorsal transverse bands (Ma et al. 2009). The result of phylogenetic tree of 14 species (including other 13 species from Penaeidae in NCBI) also indicated the close relationship between *P. semisulcatus* and *P. monodon* (Figure 1), which is consistent with the phylogenetic analyses of Penaeidae using both 16S rRNA and COI genes from mitochondrial DNA (Lavery et al. 2004). Our mitogenome data supported the sister relationship of *P. semisulcatus* and *P. monodon*. The complete mitochondrial genome sequence of *P. semisulcatus* add one more mitogenome of *Penaeus*, which will contribute to further phylogenetic and comparative mitogenome studies of *Penaeus*, and related genus.

**Figure 1.**
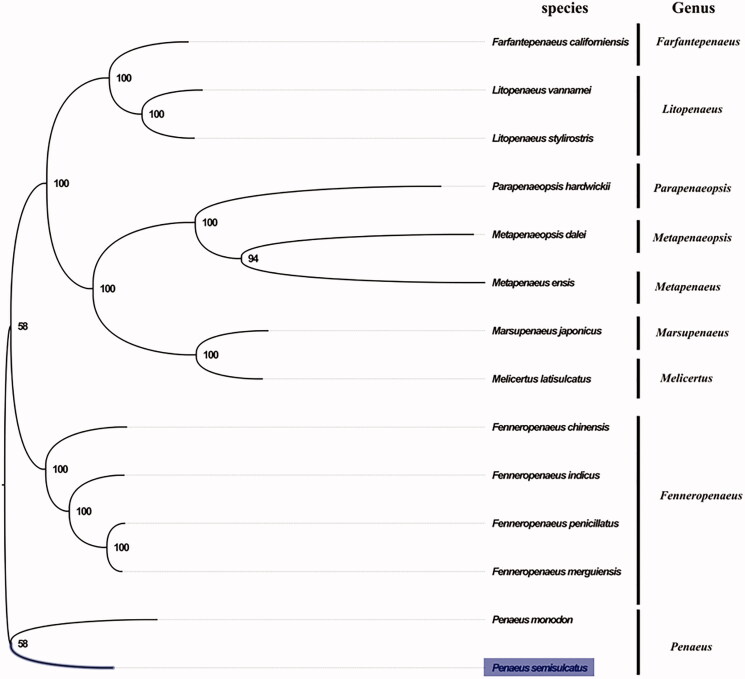
Phylogenetic tree of 14 species in family Penaeidae. The complete mitogenome is downloaded from GenBank and the phylogenic tree is constructed by maximum-likelihood method with 100 bootstrap replicates. The bootstrap values were labeled at each branch nodes. The gene’s accession number for tree construction is listed as follows: *Fenneropenaeus penicillatus* (NC_026885), *Fenneropenaeus merguiensis* (NC_026884), *Fenneropenaeus indicus* (NC_031366), *Fenneropenaeus chinensis* (NC_009679), *Penaeus monodon* (NC_002184), *Farfantepenaeus californiensis* (NC_012738), *Litopenaeus vannamei* (NC_009626), *Litopenaeus stylirostris* (NC_012060), *Marsupenaeus japonicus* (NC_007010), *Parapenaeopsis hardwickii* (NC_030277), *Metapenaeopsis dalei* (NC_029457), *Metapenaeus ensis* (NC_026834), and *Melicertus latisulcatus* (MG821353).
